# The Impact of Dietary Long-Chain Polyunsaturated Fatty Acids on Respiratory Illness in Infants and Children

**DOI:** 10.1007/s11882-012-0304-1

**Published:** 2012-09-23

**Authors:** Jeske H. J. Hageman, Pieter Hooyenga, Deborah A. Diersen-Schade, Deolinda M. Felin Scalabrin, Harry J. Wichers, Eileen E. Birch

**Affiliations:** 1 Human Nutrition Department, Wageningen University, Bornse Weilanden 9, 6708 WG Wageningen, the Netherlands; 2 Mead Johnson Nutrition, Middenkampweg 2, 6545 CJ Nijmegen, the Netherlands; 3 Mead Johnson Nutrition, Evansville, IN 47721 USA; 4 Wageningen University Food & Biobased Research, Bornse Weilanden 9, 6708 WG Wageningen, the Netherlands; 5 Retina Foundation of the Southwest, 9900 North Central Expressway, Suite 400, Dallas, TX 75231 USA

**Keywords:** Allergic rhinitis, Allergy, ARA, Arachidonic acid, Asthma, Atopic dermatitis, Atopy, Children, DHA, Docosahexaenoic acid, Eczema, Eicosapentaenoic acid, EPA, Fish, Fish oil, Immune system, Infants, LCPUFA, Maternal supplementation, Omega-3 fatty acids, Omega-6 fatty acids, Nutrition, Postnatal supplementation, Respiratory disease, Respiratory illness, Polyunsaturated fatty acids

## Abstract

Increasing evidence suggests that intake of long-chain polyunsaturated fatty acids (LCPUFA), especially omega-3 LCPUFA, improves respiratory health early in life. This review summarizes publications from 2009 through July 2012 that evaluated effects of fish, fish oil or LCPUFA intake during pregnancy, lactation, and early postnatal years on allergic and infectious respiratory illnesses. Studies during pregnancy found inconsistent effects in offspring: two showed no effects and three showed protective effects of omega-3 LCPUFA on respiratory illnesses or atopic dermatitis. Two studies found that infants fed breast milk with higher omega-3 LCPUFA had reduced allergic manifestations. Earlier introduction of fish improved respiratory health or reduced allergy in four studies. Three randomized controlled trials showed that providing LCPUFA during infancy or childhood reduced allergy and/or respiratory illness while one found no effect. Potential explanations for the variability among studies and possible mechanisms of action for LCPUFA in allergy and respiratory disease are discussed.

## Introduction

Respiratory illnesses, both infectious and allergic, are a major cause of morbidity in children. Around 95 % of children have at least one acute respiratory infection in the first 3 years of life; 75 % have upper respiratory infections (URI) [1, 2••]. Lower respiratory infections, especially pneumonia, are associated with higher mortality, being the leading cause of death in children under 5 years of age worldwide [3]. Asthma is the most prevalent chronic illness in childhood, affecting around 10 % and, in some countries, up to 25 % of children [4]. Allergic manifestations, including allergic rhinitis, asthma, and atopic dermatitis (AD, often called atopic eczema or eczema), have been increasing in the last several decades [4, 5].

Appropriate nutrition is critical early in life, when there is a window of opportunity to support normal development and function of the immune system [6–8]. The neonate has several immunological immaturities, which include priming interactions of antigen-presenting cells, cytotoxic T-cell responses to infection, complement activity, and antibody responses to antigen exposure, in addition to Th2-phenotype polarization [9]. Th1 cytokines are involved in inflammatory reactions directed to fight infections, whereas Th2 cytokines are involved in antibody production, particularly IgE, and are commonly associated with allergic reactions. Th1 and Th2 cytokines have mutually inhibitory functions [10]. The placental immunological milieu is switched towards a Th2 phenotype to ensure that the maternal immune system will not mount a Th1 response against the fetus. This also affects the newborn who is skewed towards Th2 response and needs to be redirected towards appropriate Th1 response that allows protection from infectious diseases [8]. Immunomodulatory components and nutrients in breast milk can influence the maturation of the immune system, response to infections, and development of atopic diseases; some of these nutrients may also be delivered through other dietary sources. Recently, research has focused on long-chain polyunsaturated fatty acids (LCPUFA) early in life as immunomodulatory nutrients potentially playing important roles in prevention and resolution of respiratory illnesses and allergy.

The LCPUFA of interest include the omega-3 (or n-3) LCPUFA docosahexaenoic acid (DHA, 22:6n-3) and eicosapentaenoic acid (EPA, 20:5n-3) and the omega-6 (or n-6) LCPUFA arachidonic acid (ARA, 20:4n-6). These LCPUFA are synthesized endogenously from the precursors alpha-linolenic acid (ALA, 18:3n-3) and linoleic acid (LA, 18:2n-6) through a series of elongation and desaturation steps common to the omega-3 and omega-6 pathways [11]. This conversion, particularly for ALA to DHA, is inefficient, and endogenous production will not meet the requirements of all infants during rapid tissue growth and development [12]. Research on LCPUFA early in life has focused on their roles in neurodevelopment, because DHA and ARA accumulate, especially during late pregnancy through about 2 years postnatally, in uniquely high concentrations in brain gray matter [13]. DHA, EPA, and ARA serve as important cell membrane components as well as precursors for an extensive network of biologic mediators with many effects in the body, including numerous roles in immune function and inflammation [11].

Several expert bodies recommend including specific amounts of LCPUFA in the diets of infants, children, and their mothers [14–19]. Maternal LCPUFA intake impacts delivery of these fatty acids to the infant via the placenta before birth and via breast milk [14]. Several groups recommend that pregnant and lactating women consume at least 200–250 mg DHA daily, which can be achieved by consuming fish, especially fatty fish like salmon, mackerel, and tuna, or through dietary supplements like fish or algal oil [14, 17–19]. Breast milk is the preferred sole source of nutrition for infants through 4–6 months, with continued breastfeeding to 12 months or longer. Breast milk from mothers meeting recommended DHA intakes should provide sufficient LCPUFA supply. Experts recommend that infants weaned from breastfeeding should consume a diet with adequate amounts of added DHA and ARA, in a balance similar to that typically found in breast milk [15–19], and that diets should continue to provide sufficient amounts of omega-3 LCPUFA through childhood and adulthood [17–19]. There are no specific recommendations for ARA intake by children or adults, including pregnant and nursing women, because diets are typically rich in LA, which is readily converted to ARA to meet needs.

The association between omega-3 intake from fish or fish oil supplements early in life and risk of allergy was evaluated in a systematic review of studies through 2009 [20]. It concluded that most epidemiological studies of fish intake in pregnancy, infancy, or childhood found a protective effect of fish on atopic outcomes, but the benefits of fish oil supplementation were less clear. A 2009 critical review on the use of omega-3 LCPUFA for treatment of inflammatory conditions concluded that LCPUFA may be beneficial for treatment of children with asthma [21]. In this paper, we review additional studies published from 2009 through July 2012 that examine effects of fish, fish oil or LCPUFA intake during pregnancy, lactation, and early postnatal years on allergic and infectious respiratory illnesses.

## Studies of LCPUFA and Respiratory Illness Early in Life

### Maternal LCPUFA Supply During Pregnancy and/or Lactation

A 2005 review concluded that there is evidence that providing omega-3 LCPUFA during pregnancy may reduce development or severity of allergic disease in the offspring [22]. More recent randomized controlled clinical trials (RCTs) that assessed effects of omega-3 LCPUFA or fatty fish during pregnancy and/or lactation on infant outcomes related to respiratory health, as well as studies that evaluated associations between maternal diet or breast milk fatty acids and infant allergic outcomes, are summarized in Table 1.

**Table 1:** Characteristics of studies evaluating the effects of LCPUFA during pregnancy and/or lactation on respiratory and related outcomes in infants or young children

Author, year [Ref]	Type of trial; location	Subjects	Intervention	Duration	Outcomes evaluated
Imhoff-Kunsch et al. 2011 [23•]	Double-blind RCT; Mexico	1,094 pregnant women	DHA group: 400 mg DHA from algal oil (2 capsules/day)	18–22 weeks gestation to delivery; follow-up at 1, 3, and 6 months of age	Parental reports of occurrence and duration of illness symptoms
			Control: corn/soy oil blend		
Palmer et al. 2012 [24]	Double-blind RCT; Australia	706 pregnant women expecting an infant with family history of allergy	Omega-3 group: 800 mg DHA and 100 mg EPA from fish oil (3 capsules/day)	21 weeks gestation to delivery; follow-up at 12 months of age	Diagnosis of IgE-associated allergic disease, i.e., atopic dermatitis or food allergy with sensitization (positive skin prick test to at least 1 allergen tested)
			Control: vegetable oil		
Noakes et al. 2012 [26]	RCT; United Kingdom	123 pregnant women expecting an infant with family history of allergy	Fish group: two 150 g portions of salmon per week (3.45 g EPA plus DHA)	20 weeks gestation to delivery; follow-up at 6 months of age	Cord blood fatty acids and mononuclear cell cytokine and PGE_2_ production; leukocyte phenotypes; serum total IgE at birth and 6 months; clinical outcomes at 6 months
			Control: usual diet		
Furuhjelm et al. 2009 [27]	Double-blind RCT; Sweden	145 pregnant women with allergy or husband or previous child with allergy	Omega-3 group: 1.6 g EPA and 1.1 g DHA from fish oil (9 capsules/day)	25 weeks gestation through lactation (average 3–4 months); follow-up at 3, 6, and 12 months of age	Serum IgE for specific allergens at 3 and 12 months; IgE-associated atopic dermatitis; food allergy; skin prick tests at 6 and 12 months
			Control: soy oil		
Furuhjelm et al. 2011 [28; same cohort as 27]	Double-blind RCT; Sweden	145 pregnant women with allergy or husband or previous child with allergy	Omega-3 group: 1.6 g EPA and 1.1 g DHA from fish oil (9 capsules/day)	25 weeks gestation through lactation; follow-up at 24 months of age	Serum IgE for specific allergens at 24 months; cumulative incidence (0–24 months) of positive skin prick tests, allergic symptoms, IgE-associated atopic dermatitis and other IgE-associated disease
			Control: soy oil		
Lumia et al. 2011 [29]	Retrospective observational study; Finland	Mothers with infants (*n* = 2,679) at risk of type 1 diabetes	None	Follow-up at 5 years of age	Maternal fatty acid intake by food frequency questionnaire in 8th month of pregnancy; asthma risk at 5 years
Lumia et al. 2012 [30; same population as 29]	Retrospective observational study; Finland	Mothers with infants at risk of type 1 diabetes (*n* = 1,798 pairs)	None	Follow-up at 5 years of age	Maternal fatty acid intake by food frequency questionnaire in 3rd month of lactation; asthma risk at 5 years
Thijs et al. 2011 [31]	Prospective study; the Netherlands	315 mother-infant pairs	None	Follow-up at 24 months of age	Breast milk fatty acids at 1 month postpartum; parent questionnaires on atopic outcomes; serum total IgE and IgE for specific allergens at 1 and 2 years of age
Manley et al. 2011 [32••]	Double-blind RCT; Australia	657 preterm infants whose mothers were supplemented with fish oil or placebo capsules	High DHA group: breast milk or preterm formula with 0.85–1 % fatty acids as DHA	From birth until expected date of delivery; follow-up at 18 months of age	Incidence of bronchopulmonary dysplasia; structured parenteral interviews at 12 and 18 months about medical attention/treatment for hay fever, atopic dermatitis, asthma, or food allergy and any readmissions to hospital
			Control: breast milk or standard preterm formula with 0.25–0.35 % DHA		

A large double-blind RCT of women supplemented with 400 mg DHA per day from algal oil or an LCPUFA-free placebo from 18–22 weeks gestation through parturition found that DHA reduced occurrence of parent-reported cold symptoms (cough, phlegm, nasal congestion, nasal secretion) in 1-month-old offspring [23•]. Maternal DHA supplementation also resulted in shorter duration of cough, phlegm and wheezing at 1 month, other illnesses such as ear infection and sore throat at 3 and 6 months, and nasal secretion, difficulty breathing, fever, and rash, including diaper rash and AD, at 6 months, but increased duration of rash at 1 month, nasal congestion at 3 months, and vomiting at 6 months. Overall, this trial showed that DHA supplementation in pregnancy improved respiratory health of young infants.

Another large double-blind RCT evaluated effects of fish oil supplementation, providing 800 mg DHA and 100 mg EPA daily versus placebo, from gestational week 21 until delivery to women with offspring at high risk of allergy (i.e. with a family history of allergy) [24]. There were no differences between groups in rates of respiratory tract infections, allergic manifestations, or IgE-mediated food allergies through 12 months of age. Maternal fish oil, however, resulted in lower incidence of AD and sensitization to egg. The authors note that longer follow-up would be of interest because egg allergy has been associated with respiratory allergy at later ages [25].

A recent RCT investigated whether adding fatty fish to the diet of pregnant women altered their offspring’s neonatal immune markers [26]. Women consuming less than two fatty fish servings per month and not using fish oil supplements, and whose infants were at high risk of allergy, were randomized to continue their habitual diet or add two portions of salmon weekly (providing 3.45 g EPA plus DHA) to their diet from gestational week 20 until delivery. While production of some Th1 and Th2 cytokines was lower in cord blood mononuclear cells from the salmon group, the changes did not point to a specific protective pattern against allergy or infections, and no significant differences were found in incidence or severity of AD, wheeze, bronchiolitis, or chest infections or sensitization rates of 6-month-old infants.

In another RCT, women with infants at increased allergy risk were randomized to receive a fish oil supplement providing 1.6 g EPA and 1.1 g DHA daily or placebo from gestational week 25 through 3–4 months of lactation. Prevalence of IgE-associated food allergy and AD were significantly lower at 12 months in offspring of the fish oil group compared to control [27]. During the first 2 years, incidence of IgE-associated food allergy or AD, or any IgE-associated disease, as well as sensitization to egg, were significantly lower in the fish oil group [28]. No differences were found between groups in prevalence of asthma.

In two retrospective observational studies, relationships were assessed between maternal fatty acid intake in pregnancy and in lactation and risk of asthma in 5-year-old offspring, who were at high risk of type I diabetes [29, 30]. Maternal DHA or EPA intakes at the eighth month of pregnancy or the third month of lactation were not associated with risk of childhood asthma, but lower intake of total omega-3 PUFA (driven by lower ALA) during pregnancy was. No association was seen between maternal fish intake and risk of asthma, perhaps due to low fish intake of the mothers. Low ARA intake during pregnancy was associated with decreased risk of asthma, while low ALA intake was associated with increased risk. The authors hypothesize that the reduced risk associated with low ARA intake may be explained by its role as a precursor of pro-inflammatory eicosanoids, while low ALA may result in lower synthesis of omega-3 LCPUFA, particularly EPA.

Several studies published before 2009 investigated relationships between breast milk LCPUFA and incidence of asthma or other allergic or respiratory conditions in offspring, with mixed results. One more recent study found that higher levels of the sum of omega-3 LCPUFA (DHA, EPA and docosapentaenoic acid [DPA, 22:5n-3]) in breast milk at 1 month postpartum were associated with lower prevalence of parent-reported eczema and clinically-diagnosed AD at 2 years and food sensitization to cow’s milk, egg, or peanut at 1 year [31].

An RCT of preterm infants evaluated effects of maternal supplementation with high-DHA tuna oil or placebo during lactation through expected date of delivery [32••]. Tuna oil increased breast milk DHA from 0.25 % of fatty acids to 0.85 % [33]. Bronchopulmonary dysplasia was significantly reduced in the high DHA group among boys and among all infants with birthweight <1,250 g. Risk of hay fever for boys at 12 months and for all infants and for boys at either 12 or 18 months was significantly lower in the high DHA group, but no differences were found for asthma, eczema, or food allergy.

### LCPUFA Supply Post-Weaning

When foods other than breast milk are introduced into the diet, children may receive omega-3 LCPUFA through infant or other formulas, supplements such as fish oil, or fish. Recent studies assessing effects of consumption of fish or other LCPUFA sources by infants or children on respiratory illnesses are summarized in Table 2.

**Table 2:** Characteristics of studies evaluating the effects of post-weaning consumption of LCPUFA on respiratory and related outcomes in infants or young children

Author, year (Ref)	Type of trial; location	Subjects	Intervention	Duration	Outcomes evaluated
Alm et al. 2009 [34••]	Prospective cohort study; Sweden	8,176 infants	None	Follow-up to 12 months of age	Questionnaires at 6 and 12 months of age on family, environment, food introduction, and medical symptoms including food allergy and atopic dermatitis
Goksör et al. 2011 [35; same cohort as 34••]	Prospective cohort study; Sweden	8,176 infants	None	Follow-up to 4.5 years of age	Additional questionnaires at 4.5 years of age on family, environment, feeding habits, and medical symptoms including wheezing
Alm et al. 2011 [36; same cohort as 34••]	Prospective cohort study; Sweden	8,176 infants	None	Follow-up to 4.5 years of age	Additional questionnaires at 4.5 years of age on family, environment, feeding habits, and medical symptoms including allergic rhinitis
Hesselmar et al. 2010 [37]	Prospective cohort study; Sweden	184 infants (5/6 with a history of allergy)	None	Follow-up to 18 months of age	Parent interviews of feeding practices and allergy symptoms at 6 and 12 months of age; clinical and laboratory examination for allergy diagnoses at 18 months of age
Øien et al. 2010 [38]	Prospective cohort study; Norway	3,086 infants	None	Follow-up to 2 years of age	Parent questionnaire at 1 year of age on diet and other exposure in pregnancy through 1 year, and at 2 years of age about health, especially allergic diseases
Virtanen et al. 2010 [39•]	Prospective cohort study; Finland	1,302 infants at risk of type 1 diabetes	None	Follow-up to 5 years of age	Dietary questionnaires at 3, 6, 12, and 24 months of age; questionnaire on history and symptoms of asthma, allergic rhinitis, and atopic dermatitis at 5 years of age
Birch et al. 2010 [2••]	Retrospective, cohorts from 2 double-blind RCTs; US	89 exclusively formula-fed healthy infants	DHA/ARA formula or control formula with no LCPUFA fed from first week of life	Formulas fed to 12 months of age; follow-up to 3 years of age	Medical diagnosis of atopic symptoms and respiratory infections from medical records review
D’Vaz et al. 2012 [43]	RCT (inadequate blinding); Australia	420 infants born to allergic women	Omega-3 group: 280 mg DHA and 110 mg EPA per day from fish oil	Birth to 6 months of age; follow-up to 12 months of age	Plasma and erythrocyte fatty acids and *ex vivo* immune responses to allergens at 6 months of age; allergic diseases at 6 and 12 months of age; sensitization (by skin prick test) at 12 months of age
			Control: olive oil		
Minns et al. 2010 [44•]	Double-blind RCT; US	86 healthy children 18 to 36 months of age	DHA-43: toddler formula with 43 mg algal DHA per day	Formulas fed for 60 days	Change in plasma and erythrocyte fatty acids; assessment of usual DHA intakes; adverse events including incidence of illnesses, including respiratory illnesses, from medical records and parent reports
			DHA-130: toddler formula with 130 mg algal DHA per day		
			control: toddler formula without DHA		
Thienprasert et al. 2009 [45••]	Double-blind RCT; Thailand	180 children 9 to 12 years of age	Omega-3 group: milk drink with 200 mg EPA and 1 g DHA from fish oil	Milk drinks fed 5 days per week for 6 months	Episodes and duration of illnesses during intervention; plasma phosphatidylcholine fatty acids and cytokines at end of intervention
			Control: milk drink with soy oil		

#### Fish Consumption

Associations between introduction of fish intake and atopic diseases were assessed in a large prospective cohort of children. Introduction of fish to the diet before 9 months of age had a beneficial effect on prevalence of AD [34••]. Follow-up at 4.5 years showed that early fish introduction was associated with reduced risk of recurrent wheeze [35] and allergic rhinitis [36]. A separate, smaller birth cohort study showed that earlier fish introduction was associated with lower frequency during the first 18 months for AD, food allergy, and asthma, although the latter did not reach significance [37].

Another prospective birth cohort study also found a protective effect of fish consumption, showing that more frequent fish consumption at 1 year of age was associated with reduced AD prevalence at 2 years [38]. Fish consumption did not reduce incidence of asthma, but overall prevalence of asthma, as expected, was very low at 2 years. This study did not find a protective effect of cod liver oil, suggesting that there may be protective components in fish other than LCPUFA. A prospective birth cohort study of children at high risk for type 1 diabetes found that fish introduction before 6 months reduced prevalence of allergic rhinitis at 5 years [39•], similar to an earlier study that reported that fish consumption in the first year significantly reduced the odds ratio for allergic rhinitis [40]. AD at 6 months was associated with later asthma and allergic rhinitis [39•].

#### Clinical Trials of LCPUFA Supply

Few studies have specifically investigated effects of LCPUFA addition to infant formula on respiratory illnesses and atopic diseases. Retrospective medical chart reviews were conducted through 3 years of age for infants previously participating in two double-blind RCTs [2••]. In these RCTs, infants were fed control infant formula with no LCPUFA or formula with 0.32–0.36 % of fatty acids as DHA and 0.64–0.72 % ARA through 12 months of age. The group fed DHA/ARA formula had significantly lower incidence and odds ratios for wheezing/asthma, wheezing/asthma/AD, any allergy, and URI during the first 3 years than the control group. The DHA/ARA group also had significantly shorter time to first diagnosis of wheezing/asthma, wheezing/asthma/AD, any allergy, and URI. Thus, infants fed DHA/ARA formula had delayed onset as well as lower incidence of common allergic diseases and URI. No differences were found for otitis media or combined nonallergic respiratory illnesses (URI, otitis media, sinusitis, bronchitis, bronchiolitis, and pneumonia).

Preliminary data from a very recent prospective observational study also found that infants fed an infant formula with 0.32 % DHA and 0.64 % ARA had significantly lower incidence of respiratory and allergic illnesses compared to those fed control formula with no LCPUFA [41]. This study confirms findings from an earlier observational study that showed significantly lower incidence of bronchitis/bronchiolitis at 5, 7, and 9 months of age in infants fed formula with these levels of DHA and ARA, compared to formulas with lower levels of or no LCPUFA [42]. In addition, the group fed formula with 0.32 % DHA and 0.64 % ARA had lower incidence of rhinitis at 1 month and URI at 1 and 12 months [42].

A recent double-blind RCT investigated effects of fish oil supplementation, providing 280 mg DHA and 110 mg EPA daily versus placebo, from birth until 6 months on AD and allergic sensitization in infants at high risk of allergy [43]. There were no significant effects on AD at 6 or 12 months, although there was reduced Th2 cytokine (IL-13) response to house dust mite allergen in the fish oil group, associated with lower AD at 6 and 12 months. Fish oil was also associated with a significant increase in Th1 cytokine (IFNγ and TNFα) response. This study had inadequate blinding and low adherence for the fish oil, so clinical outcomes in relationship to plasma and erythrocyte fatty acid levels were evaluated independently of allocation. This analysis showed a negative correlation between plasma DHA and development of AD, which was consistently associated with lower Th2 cytokines (IL-13 and IL-5) responses, further supporting the hypothesis that DHA sufficiency reduces risk of developing allergy.

A double-blind RCT in children 18–36 months old receiving either a milk-based toddler formula providing 130 mg (high DHA) or 43 mg algal DHA daily or a control formula without DHA for 2 months found lower incidence of respiratory illnesses, including cough, URI, pharyngitis, bronchitis, pneumonia, and strep throat, in the high DHA group [44•]; 46 % of children in the control group had at least one respiratory event, compared with 17 % in the high-DHA group. The incidence of respiratory illnesses was 41 % for the group receiving formula providing 43 mg DHA daily, which was not statistically different from the high-DHA or control groups.

Another double-blind RCT randomized 9- to 12-year-old children to receive milk with either added fish oil (providing 200 mg EPA and 1 g DHA daily, 5 days/week for 6 months) or soy oil [45••]. Fewer subjects in the group fed milk with fish oil had illnesses, of which URIs (including rhinitis, colds, and influenza) and diarrhea were most common. The fish oil group also had fewer episodes of illness per subject and shorter duration of illnesses.

## Discussion

All studies in this review evaluated possible roles for dietary omega-3 LCPUFA in immune-related outcomes, particularly related to respiratory health. Some, but not all, studies included only subjects at high risk of allergy. Dietary sources for LCPUFA, however, varied widely among studies, including fish, fish or algal oil, breast milk, and milk-based formulas with added LCPUFA. Levels of intake of specific LCPUFA also varied widely. All studies included DHA, and many included EPA, but only trials with infant formula specifically included ARA. Breast milk and fish oil also contain other LCPUFA, and fish contributes other nutrients that impact immune function. Thus, it is not possible to say what specific LCPUFA, combination of LCPUFA, source(s) or level(s) provide optimal effects on respiratory health.

Recent studies investigating increased intake of fish, fish oil, or DHA during pregnancy found inconsistent effects, with two studies showing no effects [26, 29], and three showing protective effects of omega-3 LCPUFA supply on respiratory illnesses [23•] or AD [24, 27, 28] in offspring. A 2005 review concluded that there is strong evidence that exposure to adequate omega-3 LCPUFA *in utero* and via breast milk is associated with reduced development of atopic disease in infants and children [22]. Positive findings from interventions during pregnancy cannot be ascribed exclusively to *in utero* exposure for breastfed infants, as higher LCPUFA intakes in pregnancy, especially from habitual diets, can contribute to higher LCPUFA in breast milk and thus also increase infant LCPUFA supply postnatally.

Postnatal LCPUFA intake, from breast milk, fish, fish oil, or infant formula with LCPUFA, also has potential to influence development of allergies and respiratory illnesses. While several studies found positive effects of earlier introduction of fish on respiratory allergies or other aspects of atopy [34••, 35–38, 39•], the effect could be related to factors other than omega-3 LCPUFA present in fish. Several RCTs, however, specifically assessed LCPUFA addition to infant formula and milk-based beverages for children. Two RCTs in children that evaluated dietary DHA and EPA from fish oil [45••] or DHA from algal oil [44•] found significant reductions in incidence of overall illnesses (primarily URI) or respiratory illnesses, demonstrating a benefit specific to omega-3 LCPUFA. Birch et al. [2••] found that addition of DHA in combination with ARA to infant formula has positive effects on not only URI but also allergic manifestations, including wheezing/asthma; similar results for respiratory infections and allergy were seen in two prospective observational studies [41, 42].

Higher omega-3 LCPUFA in breast milk has also been associated with less asthma, AD, and food sensitizations [31, 46, 47], and maternal supplementation during lactation reduced incidence of respiratory illnesses in preterm infants [32••]. In a cohort of infants at high risk of allergy, however, associations were found between total omega-3 LCPUFA, DPA, and DHA in colostrum and risk of sensitization to food at 6 months and to aeroallergens at 2 years of age [48, 49]. Total omega-3 and ALA in mature breast milk were associated with non-atopic eczema at 2 years, and total omega-6, LA and di-homo-γ-linolenic acid (20:3n-6) in colostrum were associated with allergic rhinitis at 7 years. One hypothesis underlying associations between DHA and ARA precursors and increased allergy might be that impaired precursor conversion to LCPUFA, especially DHA, results in a tissue DHA deficiency that may facilitate atopic manifestations.

The rate-limiting enzymes in LCPUFA synthesis are the ∆^5^ and ∆^6^ desaturases encoded by the *FADS1 FADS2* gene cluster, and some studies have found relationships between *FADS* gene variants and immune-related outcomes [50]. Variability in plasma phospholipid ARA status can be explained to a very high degree by genetic variants for *FADS 1 FADS 2* (28 % in one study), while only 1–3 % of the variability in DHA status was explained [51]; in this study, carriers of minor alleles, associated with lower levels of the LCPUFA products of the desaturases, had lower prevalence of allergic rhinitis and AD (not significant after correction for multiple testing). A recent study found that children homozygous or heterozygous for the minor allele who were exclusively breastfed for at least 3 months had lower risk of asthma than those breastfed for shorter periods, while length of breastfeeding had no effect on risk of asthma in children homozygous for the major allele [52]. In addition, women have been shown to have higher rates of DHA synthesis than men [53], which may help explain differences in immune outcomes between genders [32••].

A prevalent hypothesis links excess intake of omega-6 PUFA to increased risk for atopic disease, in part because ARA, which is present in high concentrations in cell membranes of immune cells, is the precursor for a family of eicosanoid mediators that are predominantly pro-inflammatory [20]. A recent review of fatty acid status and atopic disease, however, did not find clear support for that hypothesis, and the authors suggest that, given some reports of low ARA status associated with allergy, combining some omega-6 LCPUFA with increased omega-3 LCPUFA might prove more efficacious against atopic manifestations than omega-3 LCPUFA alone [54]. Breast milk always contains both omega-3 and omega-6 LCPUFA, albeit at variable levels and ratios, and expert recommendations for LCPUFA in infant formula specify that both DHA and ARA should be added [16, 17]. The levels and ratio for DHA and ARA are important in the infant diet because of beneficial effects on neurocognitive development [16], and this balance may also be important for the developing immune system early in life. A study in a mouse model of AD did not find any effect of ARA or DHA alone on severity of dermatitis, but found significant improvements with the 2:1 combination of ARA:DHA [55]. In preterm infants, lower blood DHA in the first month postnatally was associated with higher risk of chronic lung disease, while lower blood ARA was associated with higher risk of late-onset sepsis [56]. While the optimal levels and ratios of LCPUFA in the infant diet for immune development and respiratory health cannot be defined based on currently available data, three recent infant formula studies found that levels of DHA and ARA similar to worldwide means in breast milk resulted in significant reductions in respiratory illness and allergy early in life [2••, 16, 41, 42].

The omega-3 LCPUFA EPA and DHA can partially replace ARA in immune cell membranes, and also serve as precursors for metabolites with potent anti-inflammatory or inflammation resolution effects, and, thus, are thought to counterbalance the pro-inflammatory effects of ARA products. This is an overly simplified view, however, both with regard to the roles for omega-6 versus omega-3 LCPUFA in immune function and inflammation, as well as the mechanisms involved [11, 57]. For example, ARA gives rise to PGE_2_, which has both pro- and anti-inflammatory activity [57]. Furthermore, ARA can be metabolized into lipoxin A_4,_ an inflammation-resolving eicosanoid [58].

EPA and especially DHA have been shown to have multiple mechanisms for effects on immune function. The omega-3 LCPUFA are metabolized into resolvins and protectins with anti-inflammatory and inflammation-resolving actions [58]. For example, protectin D1 (PD1), a product of DHA shown to be present at lower levels in asthmatic patients compared to healthy individuals, reduces allergic pulmonary inflammation [59]. PD1 and resolvin D1, also derived from DHA, inhibit production of pro-inflammatory cytokines such as IL-1β, TNFα, and IFNγ [58]. EPA and DHA can inhibit activation of NF-κB, either directly via a yet unknown mechanism, or indirectly as a ligand of PPAR-γ, and further reduce production of pro-inflammatory cytokines [60, 61]. The omega-3 LCPUFA are converted into DHA- and EPA-containing endocannabinoids with anti-inflammatory activity [62], but may also decrease production of ARA-derived endocannabinoids such as anandamide, which is also immunosuppressive [63]. DHA and EPA can exert anti-inflammatory effects via G protein-coupled receptor 120 in macrophages, inhibiting TLR and TNFα pro-inflammatory signaling pathways [64]. Omega-3 LCPUFA may also impact B-cell function by increasing membrane order and the size of membrane sphingolipid/cholesterol-rich lipid rafts, with DHA showing greater effects than EPA in some models [65].

Overall, most potential mechanisms of DHA and EPA activity are associated with inhibition and/or resolution of inflammation, which is consistent with the clinical studies showing that inflammatory conditions like asthma or AD may be reduced or prevented by omega-3 LCPUFA [11]. The role of ARA in supporting inflammation necessary for host defense, in combination with active resolution of inflammation via metabolites of DHA, EPA and ARA, may help explain the lower incidence of respiratory infections when infants are fed formula with both omega-6 and omega-3 LCPUFA [2••, 41, 42].

## Conclusions

While extensive research has examined effects of omega-3 LCPUFA early in life on allergic and infectious respiratory health outcomes, there is great variability across study designs, contributing to inconsistent results. Of the recent studies reviewed, most, but not all, found beneficial effects of fish or other sources of omega-3 LCPUFA on respiratory outcomes, including reductions in asthma and other allergy manifestations or markers. Some studies, including RCTs of fish oil or DHA supplementation of pregnant women or children, as well as studies of infant formulas with DHA and ARA, also found reductions in respiratory infections. Data on the multiple mechanisms by which LCPUFA impact immune function and development provide new insights into the possible roles of LCPUFA in initiation and resolution of inflammation. Further research should be targeted to define optimal dietary sources and amounts of LCPUFA for specific age groups to support respiratory health and immune development in the neonate, throughout childhood, and beyond.

## References

[1] Harsten G, Prellner K, Heldrup J (1990). Acute respiratory tract infections in children. A three-year follow-up from birth. Acta Paediatr Scand.

[2] Birch EE, Khoury JC, Berseth CL (2010). The impact of early nutrition on incidence of allergic manifestations and common respiratory illnesses in children. J Pediatr.

[3] Liu L, Johnson HL, Cousens S (2012). Global, regional, and national causes of child mortality: an updated systematic analysis for 2010 with time trends since 2000. Lancet.

[4] Asher MI, Montefort S, Björkstén B (2006). Worldwide time trends in the prevalence of symptoms of asthma, allergic rhinoconjunctivitis, and eczema in childhood: ISAAC phases one and three repeat multicountry cross-sectional surveys. Lancet.

[5] Lee YL, Lin YC, Hwang BF, Guo YL (2005). Changing prevalence of asthma in Taiwanese adolescents: two surveys 6 years apart. Pediatr Allergy Immunol.

[6] Calder PC, Krauss-Etschmann S, de Jong EC (2006). Early nutrition and immunity - progress and perspectives. Br J Nutr.

[7] Field CJ, Johnson IR, Schley PD (2002). Nutrients and their role in host resistance to infection. J Leukoc Biol.

[8] Morein B, Blomqvist G, Hu K (2007). Immune responsiveness in the neonatal period. J Comp Pathol.

[9] Kovarik J, Siegrist CA (1998). Immunity in early life. Immunol Today.

[10] Mosmann TR, Sad S (1996). The expanding universe of T-cell subsets: Th1, Th2 and more. Immunol Today.

[11] Calder PC: Omega-3 polyunsaturated fatty acids and inflammatory processes: Nutrition or pharmacology? Br J Clin Pharmacol. 2012, In press. 10.1111/j.1365-2125.2012.04374.x PMC3575932 22765297

[12] Brenna JT, Salem N, Sinclair AJ, Cunnane SC (2009). Alpha-linolenic acid supplementation and conversion to n-3 long-chain polyunsaturated fatty acids in humans. Prostaglandins Leukot Essent Fat Acids.

[13] Martinez M (1992). Tissue levels of polyunsaturated fatty acids during early human development. J Pediatr.

[14] Brenna JT, Lapillonne A (2009). Background paper on fat and fatty acid requirements during pregnancy and lactation. Ann Nutr Metab.

[15] Uauy R, Dangour AD (2009). Fat and fatty acid requirements and recommendations for infants of 0–2 years and children of 2–18 years. Ann Nutr Metab.

[16] Hoffman DR, Boettcher JA, Diersen-Schade DA (2009). Toward optimizing vision and cognition in term infants by dietary docosahexaenoic and arachidonic acid supplementation: a review of randomized controlled trials. Prostaglandins Leukot Essent Fat Acids.

[17] French Food Safety Agency (AFSSA): Opinion of the French Food Safety Agency on the update of French population reference intakes (ANCs) for fatty acids. AFSSA; 2010: 1–9.

[18] EFSA Panel on Dietetic Products Nutrition and Allergies (NDA) (2010). Scientific opinion on dietary reference values for carbohydrates and dietary fibre. EFSA J.

[19] Food and Agriculture Organization (FAO) (2010). Fats and fatty acids in human nutrition: report of an expert consultation.

[20] Kremmyda LS, Vlachava M, Noakes PS (2011). Atopy risk in infants and children in relation to early exposure to fish, oily fish, or long-chain omega-3 fatty acids: a systematic review. Clin Rev Allergy Immunol.

[21] Galli C, Calder PC (2009). Effects of fat and fatty acid intake on inflammatory and immune responses: a critical review. Ann Nutr Metab.

[22] Dunstan JA, Prescott SL (2005). Does fish oil supplementation in pregnancy reduce the risk of allergic disease in infants?. Curr Opin Allergy Clin Immunol.

[23] Imhoff-Kunsch B, Stein AD, Martorell R (2011). Prenatal docosahexaenoic acid supplementation and infant morbidity: randomized controlled trial. Pediatrics.

[24] Palmer DJ, Sullivan T, Gold MS et al.: Effect of n-3 long chain polyunsaturated fatty acid supplementation in pregnancy on infants’ allergies in first year of life: randomised controlled trial*.* BMJ 2012;344:e184. 10.1136/bmj.e184 PMC3269207 22294737

[25] Tariq SM, Matthews SM, Hakim EA, Arshad SH (2000). Egg allergy in infancy predicts respiratory allergic disease by 4 years of age. Pediatr Allergy Immunol.

[26] Noakes PS, Vlachava M, Kremmyda LS (2012). Increased intake of oily fish in pregnancy: Effects on neonatal immune responses and on clinical outcomes in infants at 6 mo. Am J Clin Nutr.

[27] Furuhjelm C, Warstedt K, Larsson J (2009). Fish oil supplementation in pregnancy and lactation may decrease the risk of infant allergy. Acta Paediatr.

[28] Furuhjelm C, Warstedt K, Fagerås M (2011). Allergic disease in infants up to 2 years of age in relation to plasma omega-3 fatty acids and maternal fish oil supplementation in pregnancy and lactation. Pediatr Allergy Immunol.

[29] Lumia M, Luukkainen P, Tapanainen H (2011). Dietary fatty acid composition during pregnancy and the risk of asthma in the offspring. Pediatr Allergy Immunol.

[30] Lumia M, Luukkainen P, Kaila M (2012). Maternal dietary fat and fatty acid intake during lactation and the risk of asthma in the offspring. Acta Paediatr.

[31] Thijs C, Müller A, Rist L (2011). Fatty acids in breast milk and development of atopic eczema and allergic sensitisation in infancy. Allergy.

[32] Manley BJ, Makrides M, Collins CT (2011). High-dose docosahexaenoic acid supplementation of preterm infants: Respiratory and allergy outcomes. Pediatrics.

[33] Makrides M, Gibson RA, McPhee AJ (2009). Neurodevelopmental outcomes of preterm infants fed high-dose docosahexaenoic acid: a randomized controlled trial. JAMA.

[34] Alm B, Ǻberg N, Erdes L (2009). Early introduction of fish decreases the risk of eczema in infants. Arch Dis Child.

[35] Goksör E, Alm B, Thengilsdottir H (2011). Preschool wheeze - impact of early fish introduction and neonatal antibiotics. Acta Paediatr.

[36] Alm B, Goksör E, Thengilsdottir H (2011). Early protective and risk factors for allergic rhinitis at age 4 1/2 years. Pediatr Allergy Immunol.

[37] Hesselmar B, Saalman R, Rudin A (2010). Early fish introduction is associated with less eczema, but not sensitization, in infants. Acta Paediatr.

[38] Øien T, Storrø O, Johnsen R (2010). Do early intake of fish and fish oil protect against eczema and doctor-diagnosed asthma at 2 years of age? A cohort study. J Epidemiol Community Health.

[39] Virtanen SM, Kaila M, Pekkanen J (2010). Early introduction of oats associated with decreased risk of persistent asthma and early introduction of fish with decreased risk of allergic rhinitis. Br J Nutr.

[40] Nafstad P, Nystad W, Magnus P, Jaakkola JJ (2003). Asthma and allergic rhinitis at 4 years of age in relation to fish consumption in infancy. J Asthma.

[41] Scalabrin D, Mitmesser SH, Harris C. Impact of early nutrition on infectious and allergic symptoms and diseases in the first year of life. Presented at 10th Congress of the International Society for the Study of Fatty Acids and Lipids. Vancouver, Canada; May 26–30, 2012.

[42] Pastor N, Soler B, Mitmesser SH (2006). Infants fed docosahexaenoic acid- and arachidonic acid-supplemented formula have decreased incidence of bronchiolitis/bronchitis the first year of life. Clin Pediatr.

[43] D’Vaz N, Meldrum SJ, Dunstan JA (2012). Fish oil supplementation in early infancy modulates developing infant immune responses. Clin Exp Allergy.

[44] Minns LM, Kerling EH, Neely MR (2010). Toddler formula supplemented with docosahexaenoic acid (DHA) improves DHA status and respiratory health in a randomized, double-blind, controlled trial of US children less than 3 years of age. Prostaglandins Leukot Essent Fat Acids.

[45] Thienprasert A, Samuhaseneetoo S, Popplestone K (2009). Fish oil n-3 polyunsaturated fatty acids selectively affect plasma cytokines and decrease illness in Thai schoolchildren: a randomized double-blind, placebo-controlled intervention trial. J Pediatr.

[46] Wijga AH, van Houwelingen AC, Kerkhof M (2006). Breast milk fatty acids and allergic disease in preschool children: The prevention and incidence of asthma and mite allergy birth cohort study. J Allergy Clin Immunol.

[47] Hoppu U, Rinne M, Lampi AM, Isolauri E (2005). Breast milk fatty acid composition is associated with development of atopic dermatitis in the infant. J Pediatr Gastroenterol Nutr.

[48] Stoney RM, Woods RK, Hosking CS (2004). Maternal breast milk long-chain n-3 fatty acids are associated with increased risk of atopy in breastfed infants. Clin Exp Allergy.

[49] Lowe AJ, Thien FC, Stoney RM (2008). Associations between fatty acids in colostrum and breast milk and risk of allergic disease. Clin Exp Allergy.

[50] Glaser C, Lattka E, Rzehak P (2011). Genetic variation in polyunsaturated fatty acid metabolism and its potential relevance for human development and health. Matern Child Nutr.

[51] Schaeffer L, Gohlke H, Muller M (2006). Common genetic variants of the FADS1 FADS2 gene cluster and their reconstructed haplotypes are associated with the fatty acid composition in phospholipids. Hum Mol Genet.

[52] Standl M, Sausenthaler S, Lattka E (2012). FADS gene cluster modulates the effect of breastfeeding on asthma. Results from the GINIplus and LISAplus studies. Allergy.

[53] Burdge GC, Calder PC (2005). Conversion of alpha-linolenic acid to longer-chain polyunsaturated fatty acids in human adults. Reprod Nutr Dev.

[54] Sala-Vila A, Miles EA, Calder PC (2008). Fatty acid composition abnormalities in atopic disease: evidence explored and role in the disease process examined. Clin Exp Allergy.

[55] Weise C, Heunemann C, Loddenkemper C (2011). Dietary docosahexaenoic acid in combination with arachidonic acid ameliorates allergen-induced dermatitis in mice. Pediatr Allergy Immunol.

[56] Martin CR, Dasilva DA, Cluette-Brown JE (2011). Decreased postnatal docosahexaenoic and arachidonic acid blood levels in premature infants are associated with neonatal morbidities. J Pediatr.

[57] Calder PC (2009). Polyunsaturated fatty acids and inflammatory processes: New twists in an old tale. Biochimie.

[58] Serhan CN, Chiang N, Van Dyke TE (2008). Resolving inflammation: dual anti-inflammatory and pro-resolution lipid mediators. Nat Rev Immunol.

[59] Levy BD, Kohli P, Gotlinger K (2007). Protectin D1 is generated in asthma and dampens airway inflammation and hyperresponsiveness. J Immunol.

[60] van den Elsen L, Garssen J, Willemsen L (2012). Long chain N-3 polyunsaturated fatty acids in the prevention of allergic and cardiovascular disease. Curr Pharm Des.

[61] Draper E, Reynolds CM, Canavan M (2011). Omega-3 fatty acids attenuate dendritic cell function via NF-kappaB independent of PPARgamma. J Nutr Biochem.

[62] Balvers MG, Verhoeckx KC, Plastina P (2010). Docosahexaenoic acid and eicosapentaenoic acid are converted by 3T3-L1 adipocytes to N-acyl ethanolamines with anti-inflammatory properties. Biochim Biophys Acta.

[63] Cencioni MT, Chiurchiu V, Catanzaro G (2010). Anandamide suppresses proliferation and cytokine release from primary human T-lymphocytes mainly via CB2 receptors. PLoS ONE.

[64] Oh DY, Talukdar S, Bae EJ (2010). GPR120 is an omega-3 fatty acid receptor mediating potent anti-inflammatory and insulin-sensitizing effects. Cell.

[65] Rockett BD, Teague H, Harris M (2012). Fish oil increases raft size and membrane order of B cells accompanied by differential effects on function. J Lipid Res.

